# Genetic Evidence of a Recent Decline and Crossbreed Distribution of *TRPV4* c.1024G>T Variant in Domestic Cats

**DOI:** 10.1002/age.70118

**Published:** 2026-05-10

**Authors:** Hisashi Ukawa, Ayako Akashi, Hinako Hayashi, Saaya Hiyoshi‐Kanemoto, Haruka Onishi, Kai Ataka, Yuki Matsumoto

**Affiliations:** ^1^ Genetic Testing Section Anicom Pafe Inc. Yokohama Kanagawa Japan; ^2^ Research and Development Section Anicom Specialty Medical Institute Inc. Yokohama Kanagawa Japan; ^3^ Data Science Center Azabu University Sagamihara Kanagawa Japan

**Keywords:** breed‐wide distribution, domestic cat, feline genetics, genetic testing, osteochondrodysplasia, *TRPV4*

## Abstract

The folded ear phenotype of Scottish Fold cats is associated with an autosomal dominant *TRPV4* variant (c.1024G>T) linked to osteochondrodysplasia. Although genetic testing has been implemented to guide breeding, empirical evidence of its impact on allele frequency remains limited, and crossbreed investigations are lacking. Here, we evaluated longitudinal changes in *TRPV4* c.1024G>T allele frequencies in Scottish Folds and surveyed the variant in 8610 cats from 14 breeds in Japan. Overall, between 2017 and 2024, the proportion of homozygous cats significantly declined (from 14.2% to 1.9%, *p* < 0.001), whereas the frequency of heterozygous cats remained stable (39.3% vs. 51.5%, *p* > 0.74). The variant was identified primarily in Scottish Folds but was also detected in American Curls, Norwegian Forest Cats, Munchkins, and Minuets. Taken together, our results highlight that integrating *TRPV4* c.1024G>T genotyping into breeding programs can effectively reduce the prevalence of this hereditary disorder, and they warrant the expansion of genetic testing to additional breeds.

The Scottish Fold breed was first described in Scotland in the 1970s (Todd 1972; Jackson 1975). Its folded ears result from the decreased auricular cartilage elasticity and are inherited as an autosomal dominant trait (Todd 1972; Dyte and Turner 1973; Jackson 1975; Malik et al. 1999). A missense variant (c.1024G>T, p.V342F: OMIA 000319‐9685) in the *TRPV4* gene links this phenotype (Gandolfi et al. 2016; OMIA 000319‐9685). Carriers develop Scottish Fold osteochondrodysplasia (SFOCD), characterized by bone deformities and periarticular bone formation in distal limb bones and caudal vertebrae. The variant exhibits high penetrance in homozygous cats (T/T), with varying clinical severity in heterozygous individuals (G/T) (Sartore et al. 2023). To prevent homozygous cats, mating between folded‐ear individuals was discouraged (OMIA 000319‐9685). Since the identification of the causal variant, genetic testing became available and may have been increasingly used to inform breeding decisions. While screening may have reduced the variant's allele frequency, supporting data remain limited. In Japan, the use of genetic testing in companion animals has accelerated since the late 2010s (Ukawa et al. 2024). The *TRPV4* c.1024G>T variant is primarily observed in pedigreed Scottish Fold cats and non‐pedigreed Scottish Fold mixes (Anderson et al. 2022). However, the variant has also been detected in Highlander‐type cats (Anderson et al. 2022), as well as in American Curl and Munchkin individuals suspected to be outcrossed with Scottish Folds (Takanosu and Hattori 2020). Apart from comprehensive aggregate datasets (Anderson et al. 2022), few studies have specifically investigated the crossbreed distribution of the *TRPV4* c.1024G>T variant allele frequency. This study aimed to evaluate longitudinal changes and breed‐wise distribution of *TRPV4* c.1024G>T allele frequencies in Japanese purebred cats, focusing on genetic testing's impact on allele frequency and the variant spread beyond purebred Scottish Folds.

Samples were obtained from breeders and pet stores from cats born between 2017 and 2024, with 8610 animals tested across 14 breeds with > 100 individuals per breed (American Curl (*n* = 115), American shorthair (*n* = 804), Exotic (*n* = 335), Siberian (*n* = 241), Scottish Fold (*n* = 2029), Norwegian Forest (*n* = 340), British Shorthair (*n* = 744), Persian (*n* = 395), Bengal (*n* = 102), Munchkin (*n* = 999), Minuet (*n* = 556), Maine Coon (*n* = 193), Ragamuffin (*n* = 483), and Ragdoll (*n* = 1274)). Oral mucosal tissue was collected via buccal swabs, and DNA was extracted using the Chemagic DNA Buccal Swab Kit (PerkinElmer, Waltham, MA, USA) or DNAdvance Kit (Beckman Coulter, Brea, CA, USA). Samples collected between 2017 and 2022 were genotyped via Sanger sequencing following PCR amplification with KOD‐Plus v.2 polymerase (TOYOBO, Osaka, Japan). The primers used were as follows: Forward: ACCAGCCCCACATCGTC and Reverse: CCCAATCTTGCCGGTCTTGGCGGCCATC. The genomic region encompassing the *TRPV4* c.1024G>T variant was PCR amplified using primers designed based on the Felis_catus_9.0 reference genome assembly. PCR was performed under the following conditions: initial denaturation at 94°C for 2 min; 50 cycles of 94°C for 15 s, 61°C for 20 s, and 68°C for 25 s; followed by a final extension at 68°C for 5 min. Sequencing used BigDye Terminator v3.1 (Thermo Fisher Scientific, Waltham, MA, USA), analyzed on a 3730xl DNA Analyzer (Thermo Fisher Scientific). Samples from 2023 to 2024 were genotyped using an Illumina Infinium XT iSelect 96 kit (Illumina, San Diego, CA, USA), which includes a marker targeting the *TRPV4 c.1024G>T* variant. Samples that failed genotype calling in the Illumina Infinium XT iSelect 96 assay were subsequently genotyped via Sanger sequencing. Bias‐reduced multinomial logistic regression was performed to analyze *TRPV4* diploid genotypes by birth year using the *brmultinom* function in the R package brglm2 and *p*‐values were calculated by Wald tests in R (R Foundation for Statistical Computing, Vienna, Austria). The statistical significance of potential sex differences in *TRPV4* genotyping results was determined by conducting a chi‐square test for 1847 Scottish folds with available sex information, excluding individuals with unknown sex.

The proportion of cats homozygous for *TRPV4* c.1024G>T declined from 30/211 (14.2%) in 2017 to 14/730 (1.9%) in 2024. The regression analysis showed decreased homozygous cats (*β* = −0.25, standard error [SE] = 0.04, *p* < 0.001), while no change was found in heterozygous cats (*β* = −0.01, SE = 0.02, *p* = 0.74; Figure 1). No significant sex differences were observed (*p* = 0.47): homozygotes were 30/797 in males and 29/1050 in females, and heterozygotes were 385/797 and 507/1050, respectively. Previous studies have reported an association between *TRPV4* c.1024G>T and osteochondrodysplasia, with variable phenotypic expression among heterozygous cats (Gandolfi et al. 2016; Sartore et al. 2023; Rorden et al. 2021), whereas homozygous individuals consistently exhibit severe clinical signs (Gandolfi et al. 2016; Rorden et al. 2021). For animal welfare, breeding pairs with the potential to produce *TRPV4* c.1024G>T homozygous offspring should be avoided. Our results indicated a longitudinal decline in the proportion of homozygous individuals, whereas the frequency of heterozygous cats remained unchanged. The decreased frequency of homozygous cats coincided with the period during which testing for *TRPV4* c.1024G>T became available and may reflect increased uptake of genetic screening, alongside other factors influencing breeding decisions. In Japan, genetic testing in companion animals has become increasingly widespread since the late 2010s (Ukawa et al. 2024). This shift has reportedly promoted selective breeding and reduced disease‐related variants in dogs (Ukawa et al. 2024), and a similar trend may be emerging in domestic cats.

**FIGURE 1:**
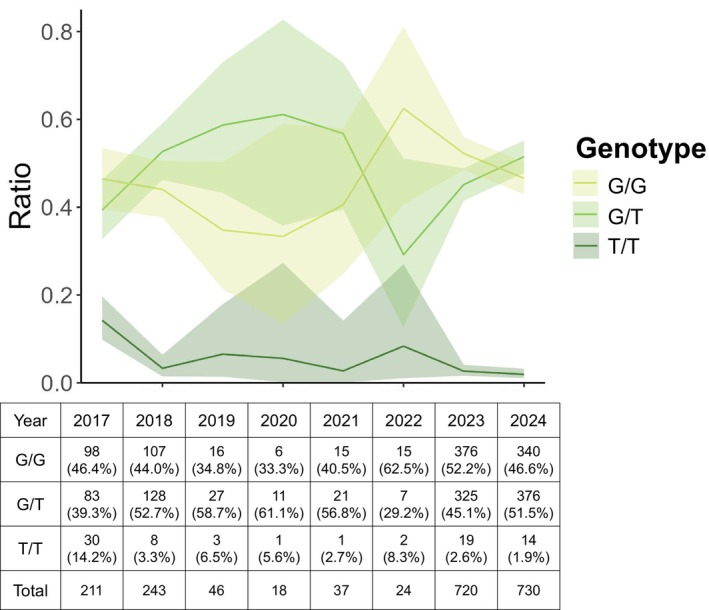
Prevalence of the *TRPV4* c.1024G>T variant by birth year in Scottish Folds. The numbers in the table indicate the number of individuals, and the values in parentheses represent the percentage for each genotype.

Breed‐wise analysis showed that homozygous and heterozygous genotypes were most frequent in Scottish Folds among 14 breeds (Figure 2). Nine breeds showed only the wild‐type genotype. Heterozygotes were detected in American Curls, Norwegian Forest, Munchkins, and Minuets, with rates of 1.7% (2/115), 0.29% (1/340), 2.8% (28/999), and 0.89% (5/556), respectively. Homozygous variants were detected in Munchkins at 0.33% (3/999). The detection of *TRPV4* c.1024G>T variant in the four breeds implies historical crossbreeding with Scottish Folds. Previous reports of *TRPV4* c.1024G>T homozygous American Curls and Munchkins exhibiting osteochondrodysplasia suggest cryptic Scottish Fold admixture within these Japanese populations (Takanosu and Hattori 2020). As the curled ear phenotype can mask the folded‐ear trait, phenotypic identification of carriers is unreliable (Takanosu and Hattori 2020; Anderson et al. 2022). Our confirmation of the variant in American Curls, Munchkins, and Minuets—including three homozygous Munchkins—highlights the importance of genetic screening in these breeds to mitigate osteochondrodysplasia risks, particularly where outcrossing is suspected, such as when curled or folded ear phenotypes are observed.

**FIGURE 2:**
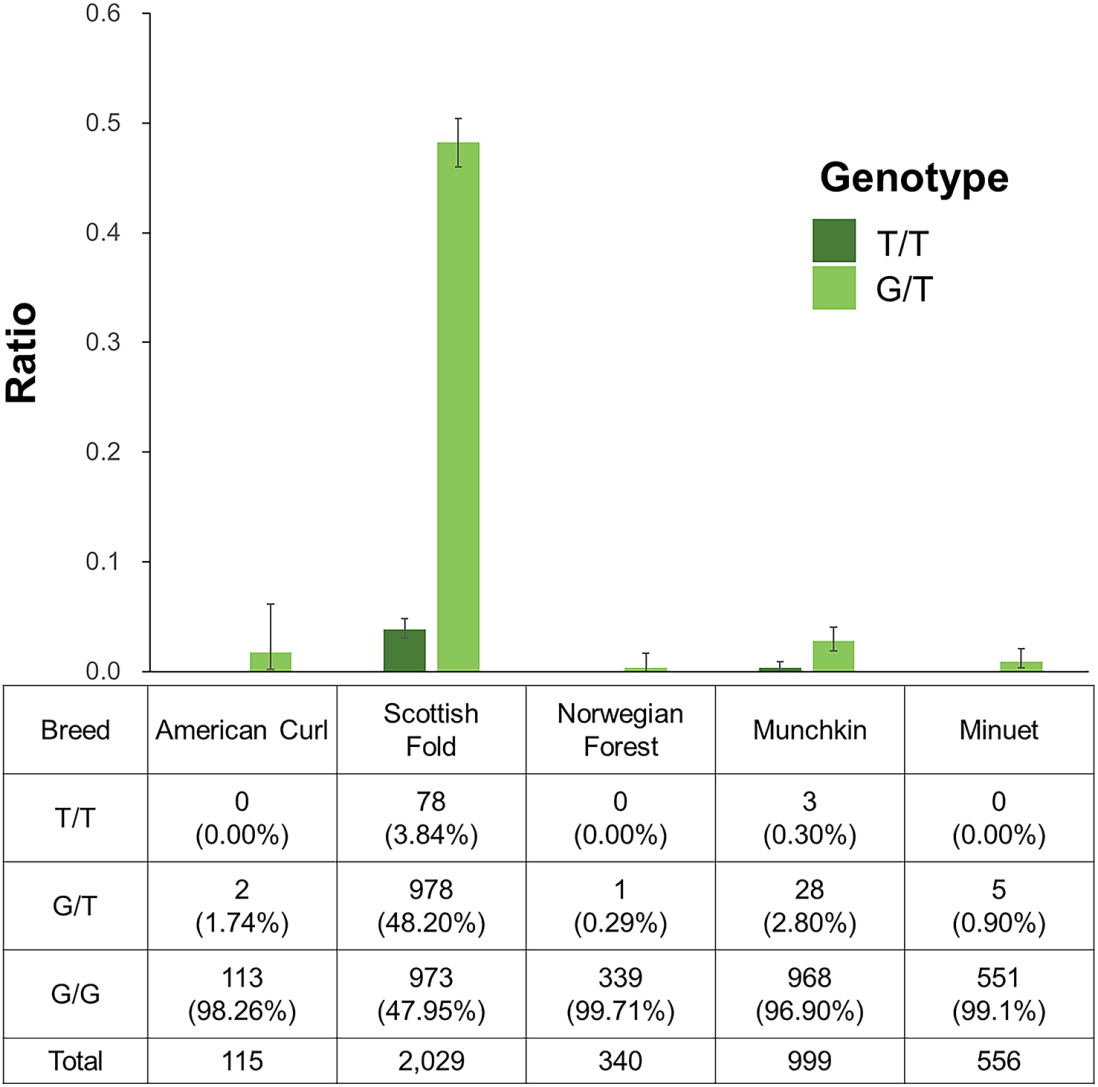
Crossbreed prevalence of the *TRPV4* c.1024G>T variant allele frequency. The numbers in the table indicate the number of individuals, and the values in parentheses represent the percentage for each genotype.

While we confirmed the variant in non‐Scottish Fold breeds, clinical associations remain unclear. Studies on American Curls and Munchkins are limited to case reports (Takanosu and Hattori 2020), and the variant impact in other breeds is uncertain. Furthermore, the results of the present study are based on Japanese cats only and given breed genetic differences across countries (Matsumoto et al. 2021), surveys elsewhere may show different outcomes. Further investigations across multiple countries are needed to evaluate *TRPV4* c.1024G>T variant associations with osteochondrodysplasia and genetic testing effects.

This study examined *TRPV4* c.1024G>T variant frequency in Scottish Folds and other breeds in Japan. Integrating *TRPV4* genotyping into breeding management may prevent variant spread and promote responsible breeding for feline welfare. While the variant exists in other breeds, its longitudinal impact and disease associations in diverse populations require further investigation. Given the ongoing debate among veterinary, breeding, and welfare communities, pursuing health‐conscious breeding and evidence‐based strategies to reduce disease risk while considering overall welfare remains important.

## Author Contributions

**Hisashi Ukawa:** data curation, formal analysis, visualization, writing – original draft, writing – review and editing. **Ayako Akashi:** data curation, formal analysis. **Hinako Hayashi:** formal analysis, writing – review and editing. **Saaya Hiyoshi‐Kanemoto:** supervision, writing – original draft, writing – review and editing. **Haruka Onishi:** supervision, writing – review and editing. **Kai Ataka:** supervision, writing – review and editing. **Yuki Matsumoto:** conceptualization, data curation, formal analysis, supervision, visualization, writing – original draft, writing – review and editing.

## Ethics Statement

All procedures were conducted in accordance with institutional, national, and international ethical guidelines. All swab samples were obtained with the consent of the owners. This study was approved by the Ethics Committee of Anicom Specialty Medical Institute Inc. (ID: 2024‐01).

## Conflicts of Interest

H.U., A.A, H.H., H.O., and K.A. are employees of Anicom Pafe Inc., a DNA testing company that offers commercial testing for the variant described in this study. S.H.‐K. is an employee of Anicom Specialty Medical Institute Inc., a sister company of Anicom Pafe Inc. Y.M. is an employee of Anicom Pafe Inc. and Anicom Specialty Medical Institute Inc.

## Data Availability

The authors have nothing to report.
